# Vascular tumors of the hand

**DOI:** 10.1186/1753-6561-9-S3-A98

**Published:** 2015-05-19

**Authors:** Caroline Leclercq

**Affiliations:** 1 Institut de la Main, 75016, Paris, France

## 

This heterogeneous group of vascular anomalies, which includes arterial, venous, capillary, and lymphatic lesions, used to be frequently referred to as “angiomas”. They have been since 1992 subdivided in two groups: vascular malformations (or “angiodysplasias”) and the rare true vascular tumours, which include immature angiomas.

## Vascular malformations

They are classified according to the type of vascular tissue affected. They may be capillary, capillovenous, venous, arterial, or lymphatic.

*Capillary angiomas* include telangiectasias (a central vivid red point with fine, spreading branches), angiokeratomas (small, dark-red structures covered by hyperkeratosis), and flat angiomas (the classic “port wine” stain), located mostly on the face and limbs. These are very difficult to treat because surgical excision leaves a significant cosmetic sequela and laser treatment may have side effects, such as pigmentation and keloid scarring.

*Capillovenous angiomas* produce a bluish subcutaneous swelling, covered by a normal skin, which may progressively increase in size. Although congenital by nature, they can develop at any age in life. On physical examination, there is neither a palpable thrill nor a murmur. Their size is variable, from a few mm^3^ to a large lesion involving several fingers.

Imaging may be necessary in the case of large lesions. Arteriogram has been supplanted by angio-MRI (or MRA), which has become an important diagnostic and therapeutic tool. Small lesions can be excised surgically. The treatment of large lesions is difficult in the hand: embolization carries a risk of digital necrosis, and surgical excision, made difficult by the lack of a plane of dissection and the number of affected pedicles, is only recommended in the localized forms.

In *angiokeratomas*, the associated skin involvement may produce dramatic cosmetic impairment. Surgical treatment is challenging in large lesions, involving skin resection and coverage by means of various plastic procedures.

*Venous angiomas* include *cavernous angioma*, which is a simple ectasia arising from a vein and can be easily removed or ligated, and dilatation *of large veins*, which is much more difficult to treat.

*Arterial angiomas* include two different entities: arterial aneurysm and arteriovenous angioma.

- Arterial aneurysms can be “false” when they arise from an hematoma resulting from arterial laceration. The lesion is saccular and histologically has neither elastic nor muscular fibres. “True” aneurysms, more commonly found in the hand, are due to a dilation of the arterial lumen. The lesion is fusiform, and its wall contains elastic and muscular fibres. In both cases, the lesion is pulsatile with a systolic thrill. The distal vascular status is assessed clinically by the pulse, and Allen’s test; and radiologically by ultrasound Doppler or an occasional MRI study. Surgical treatment consists of excision of the lesion. If there is a satisfactory collateral blood supply, microsurgical reconstruction may not be required.

- Arteriovenous malformations are abnormal communications, or shunts, between an artery and a vein which may be of traumatic or congenital origin. They may remain latent until a second injury. A venous dilation in the hand is suggestive of the diagnosis. There is a characteristic thrill, a bruit, and an increase in local temperature. Angio-MRI reveals the site of the communicating vessels and the marked, excessively rapid venous return.

Surgery must be decided on cautiously in the congenital forms of arteriovenous malformations because of the large number of shunting vessels and the possibility of other latent shunts.

*Lymphangiomas* are relatively rare. They may be superficial and circumscribed or deep. Surgery involves a risk of postoperative lymph fistula.

## True vascular tumors

***Immature angiomas*** are by far the most common vascular tumour. They appear during the first months of life. Their natural history includes a growing phase until the sixth month of life, a static phase until the twentieth month, and a slow regression phase, which may last until the sixth or seventh year and evolves to complete regression in about 90% of children. Surgery should only be undertaken at the end of the regression phase for the very rare sequelae.

Other vascular tumors are extremely rare. They include hemangioendothelioma, hemangiopericytoma, angioleiomyoma, and malignant tumors (angiosarcoma and lymphangiosarcoma).

The first two types (hemangioendothelioma and hemangiopericytoma) usually behave aggressively, and histologic differentiation with a malignant tumor is often uneasy. They require careful and frequent follow-up after surgical excision.

